# A One-Step Real-Time Multiplex PCR for Screening Y-Chromosomal Microdeletions without Downstream Amplicon Size Analysis

**DOI:** 10.1371/journal.pone.0023174

**Published:** 2011-08-22

**Authors:** Viviana Kozina, Heike Cappallo-Obermann, Jörg Gromoll, Andrej-Nikolai Spiess

**Affiliations:** 1 School of Medicine, University of Zagreb, Zagreb, Croatia; 2 Department of Andrology, University Hospital Hamburg-Eppendorf, Hamburg, Germany; 3 Center for Reproductive Medicine and Andrology, University Hospital Münster, Münster, Germany; University of Virginia, United States of America

## Abstract

**Backgound:**

Y-chromosomal microdeletions (YCMD) are one of the major genetic causes for non-obstructive azoospermia. Genetic testing for YCMD by multiplex polymerase chain reaction (PCR) is an established method for quick and robust screening of deletions in the AZF regions of the Y-chromosome. Multiplex PCRs have the advantage of including a control gene in every reaction and significantly reducing the number of reactions needed to screen the relevant genomic markers.

**Principal Findings:**

The widely established “EAA/EMQN best practice guidelines for molecular diagnosis of Y-chromosomal microdeletions (2004)” were used as a basis for designing a real-time multiplex PCR system, in which the YCMD can simply be identified by their melting points. For this reason, some AZF primers were substituted by primers for regions in their genomic proximity, and the *ZFX/ZFY* control primer was exchanged by the *AMELX/AMELY* control primer. Furthermore, we substituted the classical SybrGreen I dye by the novel and high-performing DNA-binding dye EvaGreen™ and put substantial effort in titrating the primer combinations in respect to optimal melting peak separation and peak size.

**Significance:**

With these changes, we were able to develop a platform-independent and robust real-time based multiplex PCR, which makes the need for amplicon identification by electrophoretic sizing expendable. By using an open-source system for real-time PCR analysis, we further demonstrate the applicability of automated melting point and YCMD detection.

## Introduction

In Western countries, infertility is a major health problem that affects about 15% of couples trying to conceive [1]. Y-chromosomal microdeletions (YCMD), along with Klinefelter syndrome, are the major genetic cause for primary spermatogenic failure on the male side, accounting for 5–10% of all non-obstructive azoospermic men [2].

Three regions, commonly referred to as “azoospermia factors” (AZFa, AZFb and AZFc) have been defined as spermatogenic loci and are recurrently deleted in YCMD [3], distributing among 60% (AZFc), 16% (AZFb) and 14% (AZFb+c), albeit with significant bias in different populations [4], [5]. The testicular phenotype in men bearing YCMD depends on the type of deletion, ranging from Sertoli cell-only syndrome (SCO) in AZFa [6], [7], SCO to meiotic arrest in cases of complete AZFb/AZFb+c deletions [2], up to the very heterogeneous pattern from hypospermatogenesis to SCO in AZFc deletions [8], [9].

To date, there are no defined criteria as to when patients should undergo YCMD genetic testing. An obvious target are patients subjected to intracytoplasmic sperm injection (ICSI) treatment which present with severe oligozoospermia (<1×10^6^/mL) [10]. Identification of YCMD prior to ICSI treatment can predict their transmission to offspring, as these are inevitably herited [11]. Furthermore, the extent of YCMD in a patient might be prognostic for finding spermatozoa in testicular biopsies [12]. There are also strong indications that an increased frequency of nullisomic sperm in the ejaculate of men with AZFc deletions might raise the potential risk of 45,X Turner's syndrome [13].

Consequently, genetic testing of YCMD would optimally be a routine diagnostic for the andrological laboratory, with emphasis on being relatively affordable for the consulting couple and delivering fast results for initial decision making. These demands have been solved by the development of multiplex polymerase chain reactions (PCR) using combinations of primers to simultaneously amplify sequence-tagged sites (STS) within the three genomic AZF regions, usually in combination with a non-deleted Y-chromosomal control gene such as sex-determining region Y (*SRY*) or amelogenin Y (*AMELY*). In the last years, a plethora of protocols have been developed that vary significantly in the use of selected primers, PCR chemistry and amplicon detection method. A compendium of established protocols is given in Table 1. Since the complete mapping of the male-specific region of the Y-chromosome (MSY) [14], many different STS-specific primers have been employed in the various protocols, enabling the detection of more detailed rearrangements in the palindromic/ampliconic regions of the MSY. However, for a relatively quick and versatile multiplex PCR-based YCMD detection method, it is important that the panel of STS primers is derived from regions of the MSY that are not polymorphic and that correlate well with their absence in men affected by oligo/azoospermia.

**Table 1:** Compilation of recent investigations employing single- and multiplex PCR for the detection of YCMD.

Reference	Primers	Multiplex	Detection	Real-time Dye
UMENO *et al.* [24]	classical	6-plex	Hitachi Microchip	
AKNIN-SEIFER *et al.* [41]	classical+kit	2× 6-plex	Agarose gel electrophoresis (?)	
ZHANG *et al.* [22]	selected	6-plex	Agarose/Capillary electrophoresis	SybrGreen I/FAM
LIN *et al.* [42]	partial AZFc	6-plex	Agarose gel electrophoresis	
BOR *et al.* [43]	classical	5× 5-plex	Agarose gel electrophoresis	
THORNHILL *et al.* [44]	classical	2× 4-plex	Agarose gel electrophoresis	
BOR *et al.* [25]	classical	5× 5-plex	Capillary electrophoresis	FAM/HEX
MARTINEZ *et al.* [45]	classical	3× 3-plex	Agarose gel electrophoresis	
KLEIMAN *et al.* [46]	selected for transcripts	4-, 3-, 1-plex	Agarose gel electrophoresis	
CHARPENEL *et al.* [26]	classical	4× 3-plex, 1× 4-plex	Agarose gel electrophoresis	
KENT-FIRST *et al.* [47]	classical+AZFd	9× 4-8-plex	Agarose gel electrophoresis	
HENEGARIU *et al.* [48]	classical+selected	7-, 5-, 7-, 5-, 5-plex	Agarose gel electrophoresis	
MITRA *et al.* [49]	classical+selected	2× 4-plex	Agarose gel electrophoresis	
PLASESKI *et al.* [21]	selected	11-plex	Capillary electrophoresis	FAM/HEX
FODOR *et al.* [27]	selected	5-plex	Capillary electrophoresis	
LIN *et al.* [42]	classical+selected	6-plex	Agarose gel electrophoresis	
HUCKLENBROICH *et al.* [50]	selected	3-, 2-plex	Agarose gel electrophoresis	
JABASINI *et al.* [51]	selected for haplotyping	7-, 5-plex	Hitachi Microchip	
BUCH *et al.* [23]	selected	16× 1-plex	**Melting curve**	SybrGreen I
Promega Corp., WI, USA YCMD Detection Sytem 2.0 (2009)	proprietary	6-, 5-, 4-plex	Agarose gel electrophoresis	

Primers noted are either those of Simoni *et al.* [10] (“classical”) or specifically designed for the region to be amplified (“selected”). If quantitative real-time PCRs were conducted, dye chemistry is noted under ‘Real-Time Dye’. All protocols but one (Buch *et al.* [23]) base their analysis on the electrophoretic separation of amplicons.

In this work we aimed to develop a multiplex PCR protocol which eliminates the need for downstream amplicon identification, such as agarose gel or capillary electrophoresis. On the basis of classical STS primers that cover 95% of all clinical relevant deletions reported in the literature as defined in the “EAA/EMQN guidelines for molecular diagnosis of Y-chromosomal microdeletions (2004)” [10], some of these primers were exchanged using sites in the genomic proximity. Empirically, primers and multiplex primer combinations were developed that result in a high separation of melting peaks during the melting curve program after PCR amplification, enabling in the presence of the new high-performance fluorescent dye EvaGreen™ the identification of all amplicons in a multiplex set only by means of their T_m_ values, hence making amplicon and deletion identification by amplicon size expendable.

## Results

### Validation of new AZF primer combinations

In an initial approach to develop a multiplex real-time PCR system for YCMD that utilizes only amplicon melting curve identification, we tested the classical Simoni *et al.* [10] multiplex system for its performance. We were not able to establish a functionate multiplex melting curve-based setup due to similarity of some amplicons in their melting temperature within a window of 0.5–1°C (i.e. ZFY, sY86, sY84 and sY255; see Materials & Methods), resulting in a convolution of the melting peaks that severely impeded the T_m_ identification of the amplicons. This made it necessary to evaluate other primer combinations, with the premise that substituting primers should be located in the genomic proximity of the established Simoni *et al.* [10] primers in regions that are not polymorphic and guarantee to identify over 95% of the most common AZF deletions. Using SybrGreen I chemistry, we tested over 30 different STS primers for the three different AZF regions in combinations, using the peak separability of the multiplex melting curve as the selection criterion. The classic *ZFX/ZFY* control gene, present in male and female DNA, had to be substituted by primers for *AMELX/AMELY* with 5°C less melting temperature. This made it more suitable within the framework of the remaining multiplex amplicons. The *SRY* control gene for male DNA of the Simoni *et al.* [10] setup could be included. The AZF-specific primers substituting the original ones were sY85/G34990 (instead of sY86/sY84) for the AZFa region and sY133 (instead of sY134) for the AZFb region. These primers were combined with sY254 (AZFc), sY255 (AZFc) and sY127 (AZFb) from Simoni *et al.* [10] to establish two different 4-plex setups as summarized in Table 2. High peak separability was achieved by selecting combinations in which the T_m_ values ranged from 77–87°C, with at least 2°C difference between each of the products. We had attempted to develop a 5-plex setup as in the original Simoni *et al.* [10] setup, but it was not possible to include one more amplicon delivering an additional melting peak without decreasing peak separability.

**Table 2:** Primers used for the two different multiplex setups.

Setup 1	Primers	Concentration [nM]	Origin	Amplicon Tm [°C]	Amplicon Size [bp]	Location [bp]
SRY (Control)	GAATATTCCCGCTCTCCGGA/GCTGGTGCTCCATTCTTGAG	125	Simoni et al. [10]	86.50–86.68	470	2655107–2655576
sY254 (AZFc, DAZ1-4)	GGGTGTTACCAGAAGGCAAA/GAACCGTATCTACCAAAGCAGC	50		81.51–81.83	380	25316193–25316572; 25372576–25372955; 26952264–26952643; 26986963–26987342
sY133 (AZFb)	ATTTCTCTGCCCTTCACCAG/TGATGATTGCCTAAAGGGAA	500	**NEW**	83.86–84.11	177	23499309–23499485
sY85 (AZFa)	GCTATTCTCTCTCTGGCATC-TGTATT/TGGCAATTTGCCTAT- GAAGT	375	**NEW**	76.61–76.81	80	14638102–14638181
**Setup 2**						
AMELxy (Control)	ATCAGAGCTTAAACTGGG-AAGCTG/CCCTGGGCTCTG-TAAAGAATAGTG	250	**NEW**	79.79–80.02	106 (X) 112 (Y)	11314994–11315099 (X); 6737888–6737999 (Y)
sY255 (AZFc, DAZ1-4)	GTTACAGGATTCGGCGTGAT/CTCGTCATGTGCAGCCAC	125	Simoni et al. [10]	84.35–84.52	124	25314817–25314940; 25374208–25374331; 26950888–26951011; 26988595–26988718)
sY127 (AZFb)	GGCTCACAAACGAAAAGAAA/CTGCAGGCAGTAATAAGGGA	300	Simoni et al. [10]	82.21–82.28	274	22570417–22570690
G34990 (AZFa)	CATTCGGTTTTATCAGCCAG/CAGTGACTCGAGGTTCAATG	750	**NEW**	76.41–76.56	83	15032211–15032293
**Alternatively:** AMELy (Control)	ATCAGAGCT TAAACTGGG-AAGCTG/CTCTGTAAAGAA- TAGTGGGTGGAT	500	**NEW**	79.33–79.56	105	6737888–6737992

Given are their sequences, the final concentration in the PCR reaction, the origin (either Simoni *et al.* [10] or newly designed), the amplicon melting temperatures (given as regions), amplicon size and the Y-chromosomal location based on Human Genome GRCh37/hg19 assembly (Feb. 2009).

**Figure 1:**
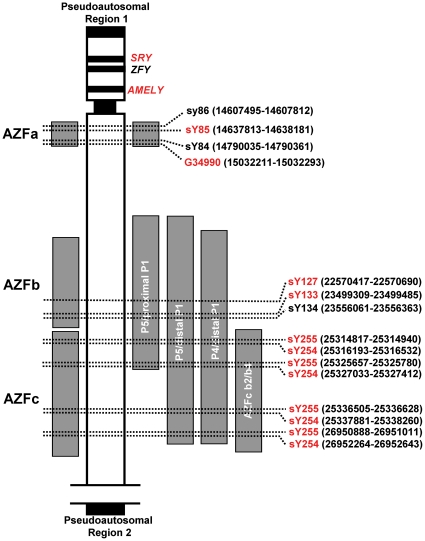
Y-chromosomal locations of the primers used for the two multiplex setups. Schematic representation of the Y-chromosome similar to Simoni *et al.* [10] showing the positions of the multiplex primers for the detection of AZFa, AZFb and AZFc deletions. The models from Vogt *et al.* [3] and Repping *et al.* [52] are shown on the left and right, respectively. The locations of the STS primers used for the two multiplex real-time setups in this work are given in red together with their genomic positions based on the GRCh37/hg19 assembly (Feb. 2009).

A detailed genomic location map of the new primers is given in Figure 1, with the primers employed in our two multiplex setups in red and substituted primers from Simoni *et al.* [10] in black. The numbers in brackets define their genomic locations based on the GRCh37/hg19 assembly (Feb. 2009).

### Increasing melting curve resolution by EvaGreen™

In principle, the two multiplex setups could be established using SybrGreen I chemistry. However, in a further effort to increase melting peak resolution, we tested two other DNA-binding dyes in respect to signal strength and peak separability. Syto-13 had been shown to be less inhibitory to PCR, show no preferential binding to GC-rich sequences and to not influence melting temperature [15]. EvaGreen™ exhibits similar features and is highly tolerable to PCR enzymes even at high concentrations, permitting strong fluorescence readouts during amplification and melting curve analysis [16].

**Figure 2:**
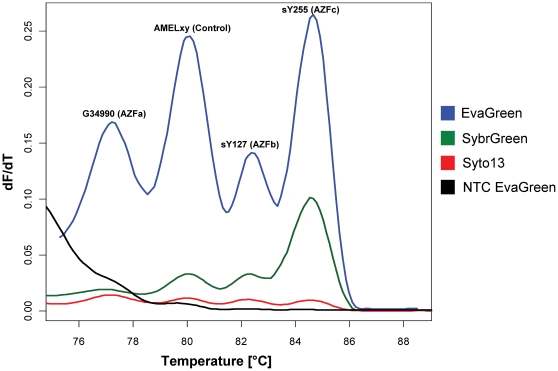
Testing three different DNA-binding fluorescent dyes for multiplex performance. EvaGreen (blue), SybrGreen (green) and Syto-13 (red) were tested for peak amplitude and peak separability using the AMELxy multiplex setup. Highest performance in respect to these two criteria was obtained with the EvaGreen dye, as can be seen by the 3–20 times higher melting peaks of the individual amplicons within the multiplex reaction compared to classical SybrGreen. NTC: baseline fluorescence of EvaGreen without DNA template.

We tested the two setups with each of these dyes and noticed an extremely superior performance of EvaGreen™, giving very high fluorescence signals during the amplification phase (data not shown) and strong signals with prominent melting peaks during the melting program, as shown with the AMELxy setup compared to the two other dyes (Figure 2). Syto-13 displayed a very low overall signal range, SybrGreen I performed better, with all (four) peaks present, but EvaGreen™ exhibited a signal range almost an order of magnitude higher. Therefore, we selected EvaGreen™ as the appropriate DNA-binding dye for YCMD detection by real-time multiplex PCR.

### Performance of the two different 4-plex setups to detect YCMD

**Figure 3:**
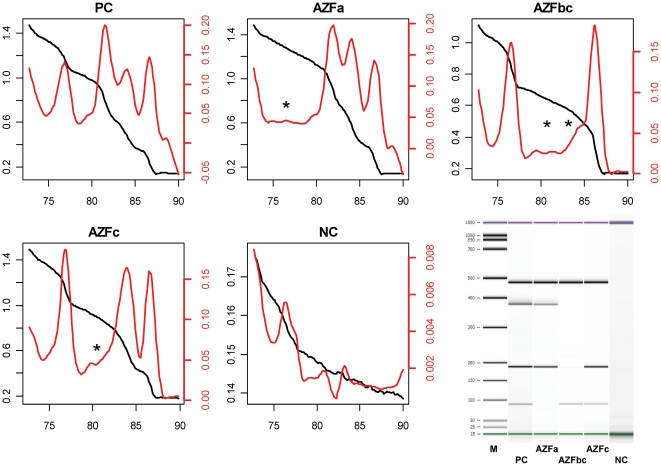
Multiplex Setup 1 (SRY). The SRY multiplex setup was tested on genomic DNA from patients without deletions (PC), AZFa deletion (AZFa), AZFb+c deletion (AZFbc), AZFc deletion (AZFc) and on a non-template control (NC). Black curves represent the original melt curve (scaled on left ordinate), while the red curves represent the first derivative melt curve (scaled on right ordinate). Asterisks denote missing peaks in comparison to the positive control. The abscissas reflect temperature [°C], ordinates reflect relative fluorescence [AU]. Bottom right: Microelectrophoretic separation (Bioanalyzer) of the amplicons obtained with the different setups as above (M = size marker).

**Figure 4:**
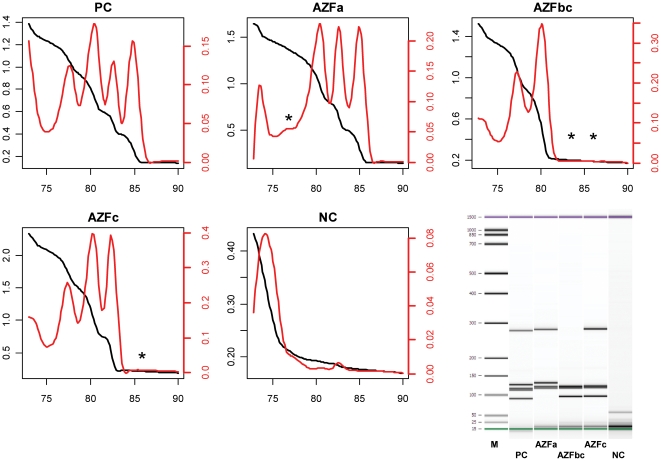
Multiplex Setup 2 (AMELxy). The AMELxy multiplex setup was tested on genomic DNA from patients without deletions (PC), AZFa deletion (AZFa), AZFb+c deletion (AZFbc), AZFc deletion (AZFc) and on a non-template control (NC). Black curves represent the original melt curve (scaled on left ordinate), while the red curves represent the first derivative melt curve (scaled on right ordinate). Asterisks denote missing peaks in comparison to the positive control. The abscissas reflect temperature [°C], ordinates reflect relative fluorescence [AU]. Bottom right: Microelectrophoretic separation (Bioanalyzer) of the amplicons obtained with the different setups as above (M = size marker).

We tested each of the two different multiplex setups on isolated genomic DNA from a fertile man (positive control: PC), and men bearing either an AZFa, AZFb+c or AZFc deletion. A non-template control (water) served for contamination testing. The results for the SRY and AMELxy multiplex setups are given in Figures 3 and 4, respectively. Each of the corresponding melting curves (black lines) exported from the PCR system was imported into the ‘qpcR’ package. The function *meltcurve* was invoked which conducts an automatic first derivative melting curve transformation (red lines) and peak identification, including calculation of the respective T_m_ values. For each of the two setups, all investigated AZF deletions could be clearly identified by the absence of the corresponding peaks, as noted by asterisks in the single melting graphs. The positive control gave four discriminable peaks in all two setups, and the control amplicons (SRY: 86.74–87.08°C; AMELxy: 79.88–79.97°C; see Table 2) were present as sharp peaks in all positive controls or AZF melting curves. We verified the amplicons that pertain to the individual melting peaks by subsequent microcapillary electrophoresis (Bioanalyzer) and could identify each of the amplicons by the corresponding present or absent bands, depending on type of AZF deletion. All T_m_ values (given as ranges) and amplicon sizes are summarized in Table 2.

### Robustness and sensitivity analysis of the two multiplex setups

To test the developed YCMD multiplex system for robustness in respect to peak strength and separability, the two setups were tested on four different genomic DNAs from men without AZF deletions. Irrespective of genomic origin, the melting peaks from all four samples could be clearly distinguished (Figure S1A). There were small individual differences in peak height/area, but in all of the samples, individual peaks were well separated. In addition, microcapillary electrophoresis displayed a homogeneous pattern of the corresponding amplicons (Figure S1B). The sensitivity of the system was evaluated by using genomic DNA from a positive control and conducting real-time PCR with subsequent melting curve analysis on increasing two-fold dilutions (Figure S1C). The individual melting peaks could be accurately identified up to an initial DNA concentration of 0.35 ng/µl after 40 cycles of amplification.

A further establishment of robustness was conducted by using 18 new samples (11 controls, 6 AZFc and 1 AZFbc) for multiplex setups performed by a different female co-worker (Figure S3, S4, S5). With the exception of one reaction (Setup 3, Con_03), in all investigated setups (Setup 1, Setup 2 and Setup 2 with alternative AMELy primer, denoted as Setup 3) four clearly separable peaks were visible for the control reactions. In agreement with the corresponding deleted regions, AZFc and AZFbc samples exhibited three or two peaks.

### Test of AMELxy control primers and portability of the multiplex setup to a 96-well block based system

In our developed multiplex system, the ZFX/ZFY control primers which are male/female specific and test for contamination with femal genomic DNA in the Simoni *et al.* [10] setup are substituted by the AMELX/AMELY primer pair in multiplex Setup 2 sharing the same features. However, in contrast to the ZFX/ZFY primers which produce a 495 bp amplicon from the X- and Y-chromosomal regions, AMELX/AMELY amplicons can be distinguished by capillary electrophoretic sizing by means of their 6 bp size difference depending on X- or Y-chromosomal origin (see double band at 106/112 bp in Figure 4). As an alternative, multiplex Setup 2 containing an AMELY-specific primer (105 bp amplicon) can be used if testing for female contamination is considered superfluous (Table 2; see Discussion). We tested the gender specificity of both primer pairs on female genomic DNA from blood and verified the AMELxy primer to amplify the female *AMELX* region, while the AMELy primer did not (Figure S2A).

As the multiplex system was established on a glass capillary-based real-time PCR system (Lightcycler™), it was important to prove general applicability by validating the performance on a 96-well block based PCR system (Stratagene MX3000). The reaction volume was extended from 20 µl to 25 µl while keeping all primer concentrations fixed at those in the initial setup (Table 2). For each of the two multiplex setups, we amplified genomic DNA from three individuals without AZF deletions, from men with AZFa/AZFb+c/AZFc deletions and a non-template control (water). Similar to our results obtained with the Lightcycler system, each of the three control samples (Figure S2B+C, upper rows) exhibited 4 clearly identifiable and separable melting peaks. In the AZFa, AZFb+c and AZFc deleted samples (Figure S2B+C, middle rows), the corresponding peaks are missing and the non-template control samples are all negative (Figure S2B+C, lower rows).

## Discussion

The establishment of YCMDs as a major cause of impaired spermatogenesis, triggered by the discovery of the *DAZ* gene [17] and the description of three discrete AZF loci [3], has led to increasing interest in the genomic analysis of male infertility. With the popularization of the PCR (polymerase chain reaction) methodology, rapid testing for the presence or absence of YCMD by amplification of the respective AZF loci is achievable in matter of a few hours. Although YCMD were initially screened by singleplex PCRs on sequence-tagged sites of the AZF regions [18], [19], [20], several multiplex PCR systems for YCMD detection have recently been developed. The simultaneous amplification of more than one AZF locus in the presence of control genes confers robustness to the interpretation of the results by ensuring a successful and sensitive amplification reaction. In light of possible false negative results when the PCR reaction fails, this avoids misinterpretations in respect to the patient's fertility status.

In addition to the plethora of existing multiplex protocols that are based on PCR amplification and subsequent analysis of the amplicons by agarose gel/capillary electrophoresis, real-time PCR approaches exist which monitor the multiplex amplification. We have compiled existing multiplex systems for the identification of YCMD in Table 1. Using real-time PCR offers the possibility of detecting initial cross-contamination, avoiding prolonged amplification in the saturation phase of the PCR, identifying low quality DNA (by high crossing points) and quantifying ratios of AZF genes or their copy numbers [21], [22]. In all established protocols, post-PCR amplicon separation by either agarose gel or capillary electrophoresis is employed to identify the presence or absence of an AZF deletion (see Table 1, ‘Detection’). We are only aware of one work in which the YCMD identification is based solely on singleplex amplicon detection by melting curve analysis [23] (see Table 1). This is an interesting approach, as the presence/absence of YCMD can be checked by the absence/presence of the corresponding amplicons, and the validity of the amplicons is quickly verified by the typical melting point (T_m_) of the PCR product. The major advantage, however, is that any following identification of the amplicons by electrophoretic size separation is made expendable, hence saving considerate time and investment.

Since a multiplex setup has several advantages over singleplex methods, such as reducing the number of reactions/input DNA and inclusion of an internal control gene for the monitoring of successful amplification, we aimed to develop a real-time based multiplex approach based only on melting curve amplicon identification. Furthermore, the method should be robust, sensitive, portable between different PCR hardwares (with little optimization needed) and give rise to automatic amplicon detection.

Similar to other investigations [23]–[26], and because there are no specific rules governing the STS marker selection, we initially chose to select the classical multiplex primer setup from Simoni *et al.* [10], covering at least 95% of all AZF loci that have been reported. As is widely established, it was possible to conduct a multiplex PCR with this setup and identify the amplicons by electrophoretic separation. However, due to the high similarity in the T_m_ values of ZFY, sY86, sY84 and sY255 (85.2°C, 86.1°C, 86.0°C, 84.7°C, respectively), an identification only by analysis of the melting peaks was not possible due to peak convolution. The substitution of the *ZFX/ZFY* control gene with the corresponding *AMELX/AMELY* control gene as in [27], sY86/sY84 by sY85/G34990 (AZFa), and sY134 by sY133 (AZFb), provided the basis for two different 4-plex setups in which the amplicon peaks displayed 2°C separability (see Table 2). The genomic proximity of the substituting primers for the AZFa and AZFb region ensures a similar outcome to the Simoni *et al.* [10] setup, as they are located within the same breakpoints [28]–[30]. However, it was not possible to include two control primer pairs in each multiplex reaction, such as *ZFX/ZFY* and *SRY* in Simoni *et al.* [10], because peak separability could not be maintained in the case of a 5-plex setup. We therefore split the multiplexes into two different 4-plex setups with SRY (male specific) and AMELxy (male/female-specific), using one control in each multiplex. With these two setups, the following scenarios can be identified: (i) Contamination with female DNA would give an increase in the 106 bp X-chromosomal derived amplicon. This is not clearly distinguishable, but the same accounts for the original Simoni *et al.* [10] setup. (ii) Accidentally using female DNA would give a negative SRY setup, and a positive AMELxy setup without AZF-specific signals. In this unlikely case, absence of male genomic DNA could be verified further by absence of the AMELy amplicon, which can be used as an alternative in Setup 2 (Table 2). (iii) In the rare case of an XX-male, the *SRY* control from Setup 1 should be positive, although recent results show that XX-males might even be negative for this gene [31]. (iv) In case of an AMELy deletion, encountered infrequently in the Asian population [32], the AMELx amplicon would serve as an internal control for a successful amplification.

The decision to exchange the commonly used SybrGreen I dye by the relatively new EvaGreen™ dye was based on the known poor performance of SybrGreen I in melting curve analysis [33], [34] and our own observation that EvaGreen™ delivered much higher fluorescence values during amplification and strong peaks during melting curve analysis. We then put considerable effort in titrating the primers with the goal of obtaining fairly equal amounts of the individual amplicons [35], an essential prerequisite for robust peak identification.

With this developed multiplex system, we were able to accurately identify the missing amplicons as a consequence of YCMD deletions in genomic DNA from men with AZFa, AZFb+c and AZFc deletions. The identification of the YCMD was robust, sensitive and independent of PCR hardware. For the safe-minded, and as shown by the microelectrophoretic separations of the multiplex PCRs in this work, it is possible for an initial validation (i.e. when setting up this system in a laboratory) to correlate and validate the melting peaks from the PCR machine with the electrophoretic sizing of the amplicons. But we want to emphasize that this is only necessary once, as the T_m_ values alone suffice. Furthermore, by using the ‘qpcR’ package for the open-source statistical environment *R*, we show in this work the potential for automatic peak identification of the multiplex melting curves, including T_m_ calculation.

In recent years, other genetic alterations have been identified which are involved in male infertility, such as Y-chromosomal haplogroups that are associated with reduced fertility [36], partial deletions in the AZFc region [37] or point-mutations in AZF located genes [38]. One drawback of our multiplex system, which is restricted by the number of amplicons whose melting peaks can be clearly distinguished, is that it can only identify larger deletions, as in the well established Simoni *et al.* [10] protocol.

We believe that our method is a great improvement to existing multiplex PCR methods for the detection of YCMD, since it is the first real-time based multiplex approach in this field, with all advantages of the latter such as amplification monitoring, an internal control in each reaction, decreased PCR setup number and most importantly, the elimination of any downstream amplicon analysis such as gel/capillary electrophoresis. A potential automation of T_m_ and hence YCMD identification, as exemplified in this work, may increase the benefit of this system even further.

## Materials and Methods

### Isolation of genomic DNA

Genomic DNA from control (n = 5), AZFa (n = 1), AZFbc (n = 4) and AZFc (n = 7) samples was isolated from testicular tissue obtained from a therapeutic testicular sperm extraction procedure (TESE), after direct surgical immersion of a rice grain sized biopsy sample in 2 ml RNAlater® (Ambion, Austin, TX), using the DNeasy Tissue Kit (Qiagen, Hilden, Germany). A second set of control (n = 11), AZFc (n = 6) and AZFbc (n = 1) samples was additionally investigated by a different female scientist to test against individual bias. Written informed consent and approval was obtained from the Ethical Commision of the Medical Board Hamburg (Approval OB/X/2000), and the studies conducted in accordance with the guidelines of the *Helsinki Declaration* regarding the use of human tissues. For more details on this procedure, see [39]. DNA purity (ratio 260 nm/280 nm) was assessed on an Ultrospec 3000 spectrophotometer (GE Healthcare, Freiburg Germany). Genomic DNA integrity was checked by 1% agarose/TAE gelelectrophoresis. Female DNA from blood was obtained with the DNeasy Tissue Kit (Qiagen) according to the manufacturer's protocol. Genomic DNA from a male with an AZFa deletion was isolated from blood with the FlexiGene DNA Kit (Qiagen).

### Selection of suitable multiplex primers for the detection of YCMD

From an empirical (by quantitative PCR) validation of over 30 primer pairs deposited in the NCBI UniSTS database (http://www.ncbi.nlm.nih.gov/unists/), we selected primer combinations which resemble a similar setup as in the widely established Simoni *et al.* protocol [10]. However, the classical setup is only applicable when subsequent amplicon size analysis by gel electrophoresis is conducted. The amplicons can not be discriminated by melting curve analysis alone as their T_m_ values differ only in a region of 0.5–1°C. This was especially the case for primers specific to ZFY (control gene, T_m_ = 85.2°C), sY86 (AZFa, T_m_ = 86.1°C), sY84 (AZFa, T_m_ = 86.0°C) and sY255 (AZFc, T_m_ = 84.7°C) whose combined amplicons lacked separability in melting curve analysis. Using the NCBI UniSTS database, alternative primers were selected for genomic sites in near proximity to the original primers. These were validated through quantitative PCR by combining them with any of the remaining primers to establish maximal separability of the melting curve peaks (T_m_ values). Primer sequences were rechecked for sequence specificity by NCBI BLAST (http://blast.ncbi.nlm.nih.gov/) and UCSC Genome ePCR (http://genome.ucsc.edu/cgi-bin/hgPcr?command=start). Validated substitutes for the primers above which displayed high separability in their T_m_ values were AMELxy (control), sY85 (AZFa), G34990 (AZFa), and sY133 (AZFb). These primers were combined with the remaining primers sY254 (AZFc), sY255 (AZFc) and sY127 (AZFb) from Simoni *et al.* [10] to establish two different 4-plex setups as summarized in Table 2.

### Quantitative real-time PCR (qPCR) setup and parameters

The two different multiplex setups were tested with three different DNA-binding dyes (SybrGreen I, Invitrogen, Darmstadt, Germany; Syto-13, Invitrogen, Darmstadt, Germany; EvaGreen™, Biotium, Hayward, USA) in order to investigate dye-dependent peak resolution during melting curve analysis. Quantitative real-time PCR was conducted in a Lightcycler™ 1.0 instrument (Roche, Basel, Switzerland) in glass capillaries at 20 µl volume containing 1× Premix Ex TaqTM (Takara Bio Europe, France) with either 0.5× SybrGreen I (from 10000× stock), 2.5 µM Syto-13 (from 5 mM stock) or 0.2× EvaGreen (from 20× stock) and optimally titrated (with respect to equal melting peak area) final primer concentrations in the PCR reactions as indicated in Table 2. PCR cycling was conducted with an initial denaturation at 95°C for 1 min, 35 cycles with 95°C 10 s/60°C 30 s/72°C 30 s, and final cooling to 40°C for 1 min. A melting curve was run after cycling by an initial ramping from 40°C to 70°C at 2°C/s and a subsequent higher resolution melting program from 70°C to 95°C at 0.1°C/s. The raw fluorescence melting curve data (temperature versus fluorescence) was exported from the Lightcycler v3.5 software. To extend applicability of our method from a capillary-based real-time PCR system to a block-based (96-well) system, PCR reactions with setups similar to above but in 25 µl volume were conducted on a Stratagene Mx3000P PCR instrument (Agilent Technologies, Palo Alto, CA, USA) and the melting data exported by the accompanying software.

### Melting curve analysis, automatic peak detection and T_m_ identification

Exported raw fluorescence melting curve data was imported into the ‘qpcR’ package [40] for the open-source statistical environment *R* (www.r-project.org). The function *meltcurve*, which provides automatic raw fluorescence data smoothing, first derivative calculation and automatic peak detection with T_m_ identification, was used to analyze the raw melt data and identify present or absent amplicons in control and AZF samples. All melting curves in this work were made by the corresponding *plot* parameter of this function.

### Validation of amplicons by microcapillary electrophoresis

To verify the validity of the multiplex amplicons, PCR reactions were size-separated after cycling on an Agilent Bioanalyzer (Model 2100; Agilent Technologies, Palo Alto, CA, USA) using the DNA 1000 capillary chip and checked for their absence in AZF deleted samples.

## Supporting Information

Figure S1 **Robustness and sensitivity analysis of the two multiplex setups.** (A) The two different multiplex setups were tested on four different genomic DNAs from patients without AZF deletions. Independent of DNA origin, the melting curves display a highly similar pattern. (B) Microelectrophoretic (Bioanalyzer) separation of the amplicons obtained in (A). (C) Testing of the two multiplex setups on a positive control (no AZF deletion) on six 2-fold dilutions. The DNA concentrations are given in the legend on the right side. Robust melting patterns were obtained with DNA concentrations higher than 0.35 ng/µl.
(TIF)

Figure S2 **Test of new AMELxy/AMELy control primers for gender specificity and evaluation of the two multiplex setups on a block-based (96-well) quantitative PCR system (Stratagene 3000).** (A) Female genomic DNA extracted from blood was subjected to quantitative PCR using the AMELxy and AMELy (alternative) primer pairs included in the multiplex setups. As expected, AMELxy gave a positive signal due to homology with the AMELx gene, while AMELy was negative. (B) and (C) Evaluation of the SRY and AMELxy multiplex setup on a 96-well quantitative PCR system (Stratagene 3000), respectively. Each of the setups was tested on three positive controls (genomic DNA from men without YCMD, upper row), samples from men with AZFa, AZFbc and AZFc-deletions (middle row) and negative control (lower row).
(TIF)

Figure S3 **Test for robustness of Setup 1 by using a second set of control (n = 11), AZFc (n = 6) and AZFbc (n = 1) samples (Lightcycler 1.0).** Setup 1 was tested on an independent set of control (Con_01–Con_11), AZFc (AZFc_01–AZFc_06) and AZFbc (AZFbc_01) samples by a different female scientist. Melting curves of control samples displayed clear visibility of four peaks. Correspondingly, AZFc and AZFbc samples exhibited three or two peaks, respectively. Bottom right: Boxplot from the melting points (T_m_ values) of all amplicons.
(TIF)

Figure S4 **Test for robustness of Setup 2 by using a second set of control (n = 11), AZFc (n = 6) and AZFbc (n = 1) samples (Lightcycler 1.0).** Setup 2 was tested on an independent set of control (Con_01–Con_11), AZFc (AZFc_01–AZFc_06) and AZFbc (AZFbc_01) samples by a different female scientist. Melting curves of control samples displayed clear visibility of four peaks. Correspondingly, AZFc and AZFbc samples exhibited three or two peaks, respectively. Bottom right: Boxplot from the melting points (T_m_ values) of all amplicons.
(TIF)

Figure S5 **Test for robustness of Setup 3 by using a second set of control (n = 11), AZFc (n = 6) and AZFbc (n = 1) samples (Lightcycler 1.0).** Setup 3 (Setup 2 with alternative AMELy primer) was tested on an independent set of control (Con_01–Con_11), AZFc (AZFc_01–AZFc_06) and AZFbc (AZFbc_01) samples by a different female scientist. Melting curves of control samples displayed clear visibility of four peaks. Correspondingly, AZFc and AZFbc samples exhibited three or two peaks, respectively. With the exception of one run (Con_03), all melting peaks could be easily distinguished. Bottom right: Boxplot from the melting points (T_m_ values) of all amplicons.
(TIF)

## References

[1] de Kretser DM (1997). Male infertility.. Lancet.

[2] Foresta C, Moro E, Ferlin A (2001). Y chromosome microdeletions and alterations of spermatogenesis.. Endocr Rev.

[3] Vogt PH, Edelmann A, Kirsch S, Henegariu O, Hirschmann P (1996). Human Y chromosome azoospermia factors (AZF) mapped to different subregions in Yq11.. Hum Mol Genet.

[4] Cram DS, Osborne E, McLachlan RI (2006). Y chromosome microdeletions: implications for assisted conception.. Med J Aust.

[5] Simoni M, Tuttelmann F, Gromoll J, Nieschlag E (2008). Clinical consequences of microdeletions of the Y chromosome: the extended Munster experience.. Reprod Biomed Online.

[6] Foresta C, Moro E, Rossi A, Rossato M, Garolla A (2000). Role of the AZFa candidate genes in male infertility.. J Endocrinol Invest.

[7] Hopps CV, Mielnik A, Goldstein M, Palermo GD, Rosenwaks Z (2003). Detection of sperm in men with Y chromosome microdeletions of the AZFa, AZFb and AZFc regions.. Hum Reprod.

[8] Reijo R, Alagappan RK, Patrizio P, Page DC (1996). Severe oligozoospermia resulting from deletions of azoospermia factor gene on Y chromosome.. Lancet.

[9] Oates RD, Silber S, Brown LG, Page DC (2002). Clinical characterization of 42 oligospermic or azoospermic men with microdeletion of the AZFc region of the Y chromosome, and of 18 children conceived via ICSI.. Hum Reprod.

[10] Simoni M, Bakker E, Krausz C (2004). EAA/EMQN best practice guidelines for molecular diagnosis of y-chromosomal microdeletions. State of the art 2004.. Int J Androl.

[11] Kent-First MG, Kol S, Muallem A, Ofir R, Manor D (1996). The incidence and possible relevance of Y-linked microdeletions in babies born after intracytoplasmic sperm injection and their infertile fathers.. Mol Hum Reprod.

[12] Brandell RA, Mielnik A, Liotta D, Ye Z, Veeck LL (1998). AZFb deletions predict the absence of spermatozoa with testicular sperm extraction: preliminary report of a prognostic genetic test.. Hum Reprod.

[13] Foresta C, Garolla A, Bartoloni L, Bettella A, Ferlin A (2005). Genetic abnormalities among severely oligospermic men who are candidates for intracytoplasmic sperm injection.. J Clin Endocrinol Metab.

[14] Skaletsky H, Kuroda-Kawaguchi T, Minx PJ, Cordum HS, Hillier L (2003). The male-specific region of the human Y chromosome is a mosaic of discrete sequence classes.. Nature.

[15] Gudnason H, Dufva M, Bang DD, Wolff A (2007). Comparison of multiple DNA dyes for real-time PCR: effects of dye concentration and sequence composition on DNA amplification and melting temperature.. Nucleic Acids Res.

[16] Mao F, Leung WY, Xin X (2007). Characterization of EvaGreen and the implication of its physicochemical properties for qPCR applications.. BMC Biotechnol.

[17] Reijo R, Lee TY, Salo P, Alagappan R, Brown LG (1995). Diverse spermatogenic defects in humans caused by Y chromosome deletions encompassing a novel RNA-binding protein gene.. Nat Genet.

[18] Simoni M, Gromoll J, Dworniczak B, Rolf C, Abshagen K (1997). Screening for deletions of the Y chromosome involving the DAZ (Deleted in AZoospermia) gene in azoospermia and severe oligozoospermia.. Fertil Steril.

[19] Fujisawa M, Shirakawa T, Kanzaki M, Okada H, Arakawa S (2001). Y-chromosome microdeletion and phenotype in cytogenetically normal men with idiopathic azoospermia.. Fertil Steril.

[20] Kim SW, Kim KD, Paick JS (1999). Microdeletions within the azoospermia factor subregions of the Y chromosome in patients with idiopathic azoospermia.. Fertil Steril.

[21] Plaseski T, Noveski P, Trivodalieva S, Efremov GD, Plaseska-Karanfilska D (2008). Quantitative fluorescent-PCR detection of sex chromosome aneuploidies and AZF deletions/duplications.. Genet Test.

[22] Zhang J, Li PQ, Yu QH, Chen HY, Li J (2008). Development of a multiplex quantitative fluorescent PCR assay for identification of rearrangements in the AZFb and AZFc regions.. Mol Hum Reprod.

[23] Buch B, Galan JJ, Lara M, Ruiz R, Segura C (2003). Scanning of Y-chromosome azoospermia factors loci using real-time polymerase chain reaction and melting curve analysis.. Fertil Steril.

[24] Umeno M, Shinka T, Sato Y, Yang XJ, Baba Y (2006). A rapid and simple system of detecting deletions on the Y chromosome related with male infertility using multiplex PCR.. J Med Invest.

[25] Bor P, Hindkjaer J, Kolvraa S, Ingerslev HJ (2003). A new approach for screening for Y microdeletions: capillary electrophoresis combined with fluorescent multiplex PCR.. J Assist Reprod Genet.

[26] Charpenel C, Guillon AS, Dorson O, Boucly C, Mathieu B (2002). A simplified method for the detection of Y chromosome microdeletions in infertile men using a multiplex sequence-tagged site-based amplification.. Genet Test.

[27] Fodor F, Kamory E, Csokay B, Kopa Z, Kiss A (2007). Rapid detection of sex chromosomal aneuploidies by QF-PCR: application in 200 men with severe oligozoospermia or azoospermia.. Genet Test.

[28] Costa P, Goncalves R, Ferras C, Fernandes S, Fernandes AT (2008). Identification of new breakpoints in AZFb and AZFc.. Mol Hum Reprod.

[29] Sun C, Skaletsky H, Rozen S, Gromoll J, Nieschlag E (2000). Deletion of azoospermia factor a (AZFa) region of human Y chromosome caused by recombination between HERV15 proviruses.. Hum Mol Genet.

[30] Kamp C, Hirschmann P, Voss H, Huellen K, Vogt PH (2000). Two long homologous retroviral sequence blocks in proximal Yq11 cause AZFa microdeletions as a result of intrachromosomal recombination events.. Hum Mol Genet.

[31] Rajender S, Rajani V, Gupta NJ, Chakravarty B, Singh L (2006). SRY-negative 46,XX male with normal genitals, complete masculinization and infertility.. Mol Hum Reprod.

[32] Jobling MA, Lo IC, Turner DJ, Bowden GR, Lee AC (2007). Structural variation on the short arm of the human Y chromosome: recurrent multigene deletions encompassing Amelogenin Y.. Hum Mol Genet.

[33] Giglio S, Monis PT, Saint CP (2003). Demonstration of preferential binding of SYBR Green I to specific DNA fragments in real-time multiplex PCR.. Nucleic Acids Res.

[34] Varga A, James D (2006). Real-time RT-PCR and SYBR Green I melting curve analysis for the identification of Plum pox virus strains C, EA, and W: effect of amplicon size, melt rate, and dye translocation.. J Virol Methods.

[35] Henegariu O, Heerema NA, Dlouhy SR, Vance GH, Vogt PH (1997). Multiplex PCR: critical parameters and step-by-step protocol.. Biotechniques.

[36] Krausz C, Quintana-Murci L, Rajpert-De Meyts E, Jorgensen N, Jobling MA (2001). Identification of a Y chromosome haplogroup associated with reduced sperm counts.. Hum Mol Genet.

[37] Moro E, Ferlin A, Yen PH, Franchi PG, Palka G (2000). Male infertility caused by a de novo partial deletion of the DAZ cluster on the Y chromosome.. J Clin Endocrinol Metab.

[38] Sun C, Skaletsky H, Birren B, Devon K, Tang Z (1999). An azoospermic man with a de novo point mutation in the Y-chromosomal gene USP9Y.. Nat Genet.

[39] Feig C, Kirchhoff C, Ivell R, Naether O, Schulze W (2007). A new paradigm for profiling testicular gene expression during normal and disturbed human spermatogenesis.. Mol Hum Reprod.

[40] Ritz C, Spiess AN (2008). qpcR: an R package for sigmoidal model selection in quantitative real-time polymerase chain reaction analysis.. Bioinformatics.

[41] Aknin-Seifer IE, Touraine RL, Lejeune H, Laurent JL, Lauras B (2003). A simple, low cost and non-invasive method for screening Y-chromosome microdeletions in infertile men.. Hum Reprod.

[42] Lin YW, Hsu CL, Yen PH (2006). A two-step protocol for the detection of rearrangements at the AZFc region on the human Y chromosome.. Mol Hum Reprod.

[43] Bor P, Hindkjaer J, Ingerslev HJ, Kolvraa S (2001). Multiplex PCR for screening of microdeletions on the Y chromosome.. J Assist Reprod Genet.

[44] Thornhill AR, Guenther AJ, Barbarotto GM, Session DR, Damario MA (2002). False-positive Y-microdeletion result for a fertile male caused by an alteration under a PCR primer.. Int J Androl.

[45] Martinez MC, Bernabe MJ, Gomez E, Ballesteros A, Landeras J (2000). Screening for AZF deletion in a large series of severely impaired spermatogenesis patients.. J Androl.

[46] Kleiman SE, Yogev L, Hauser R, Botchan A, Maymon BB (2007). Expression profile of AZF genes in testicular biopsies of azoospermic men.. Hum Reprod.

[47] Kent-First M, Muallem A, Shultz J, Pryor J, Roberts K (1999). Defining regions of the Y-chromosome responsible for male infertility and identification of a fourth AZF region (AZFd) by Y-chromosome microdeletion detection.. Mol Reprod Dev.

[48] Henegariu O, Hirschmann P, Kilian K, Kirsch S, Lengauer C (1994). Rapid screening of the Y chromosome in idiopathic sterile men, diagnostic for deletions in AZF, a genetic Y factor expressed during spermatogenesis.. Andrologia.

[49] Mitra A, Dada R, Kumar R, Gupta NP, Kucheria K (2008). Screening for Y-chromosome microdeletions in infertile Indian males: utility of simplified multiplex PCR.. Indian J Med Res.

[50] Hucklenbroich K, Gromoll J, Heinrich M, Hohoff C, Nieschlag E (2005). Partial deletions in the AZFc region of the Y chromosome occur in men with impaired as well as normal spermatogenesis.. Hum Reprod.

[51] Jabasini M, Ewis AA, Xu F, Mohamadi MR, Ping G (2005). Multiplex PCR with multichannel microchip electrophoresis: an ultrafast analysis for genetic diseases.. J Chromatogr Sci.

[52] Repping S, Skaletsky H, Lange J, Silber S, Van Der Veen F (2002). Recombination between palindromes P5 and P1 on the human Y chromosome causes massive deletions and spermatogenic failure.. Am J Hum Genet.

