# Supplementary figures and images for: A One-Step Real-Time Multiplex PCR for Screening Y-Chromosomal Microdeletions without Downstream Amplicon Size Analysis
Source: PLoS One. 2011 Aug 22;6(8):e23174. doi: 10.1371/journal.pone.0023174 (PMC3161745; doi:10.1371/journal.pone.0023174)

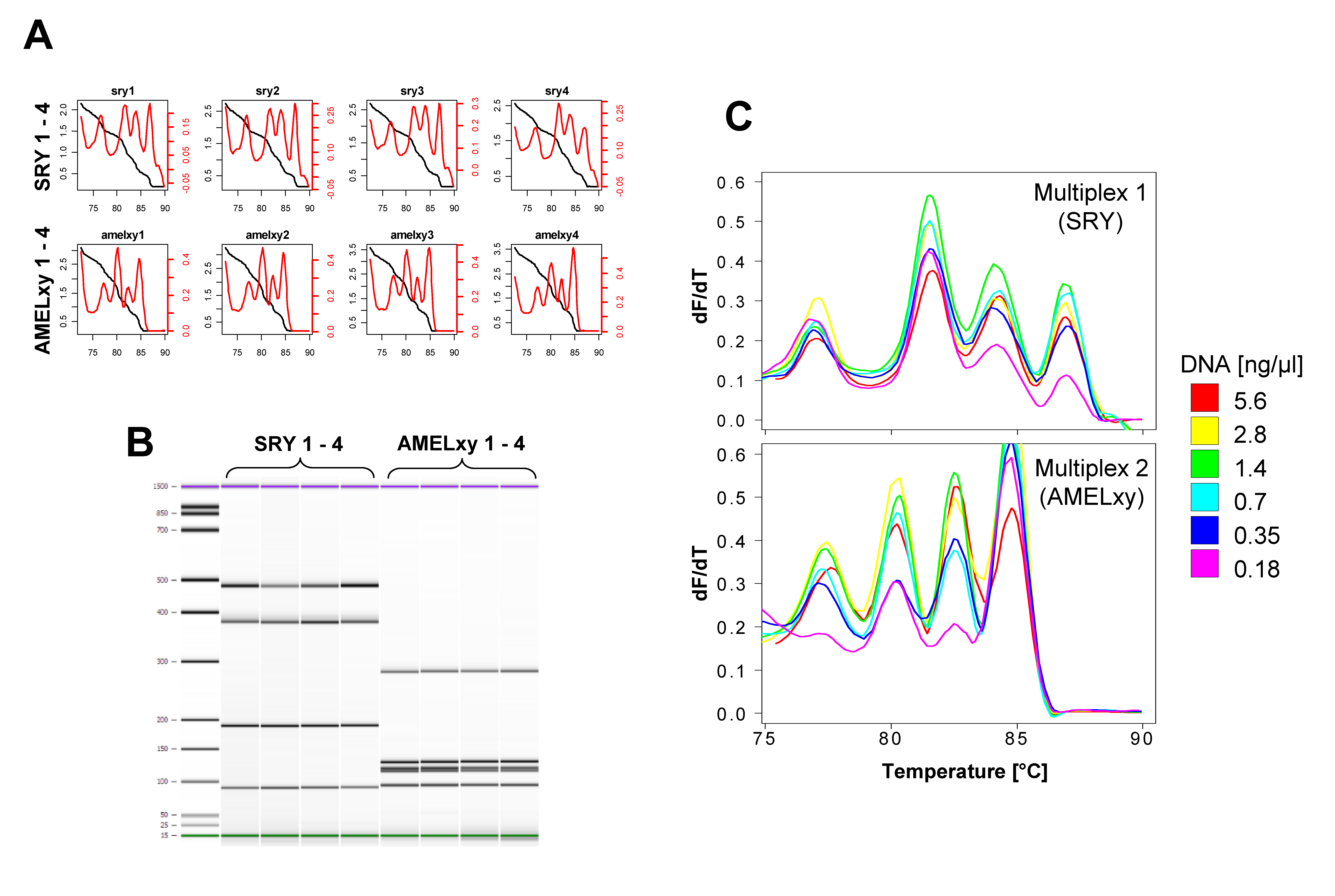

Supplement: Figure S1 — Robustness and sensitivity analysis of the two multiplex setups. (A) The two different multiplex setups were tested on four different genomic DNAs from patients without AZF deletions. Independent of DNA origin, the melting curves display a highly similar pattern. (B) Microelectrophoretic (Bioanalyzer) separation of the amplicons obtained in (A). (C) Testing of the two multiplex setups on a positive control (no AZF deletion) on six 2-fold dilutions. The DNA concentrations are given in the legend on the right side. Robust melting patterns were obtained with DNA concentrations higher than 0.35 ng/µl. (TIF) [file pone.0023174.s001.tif]

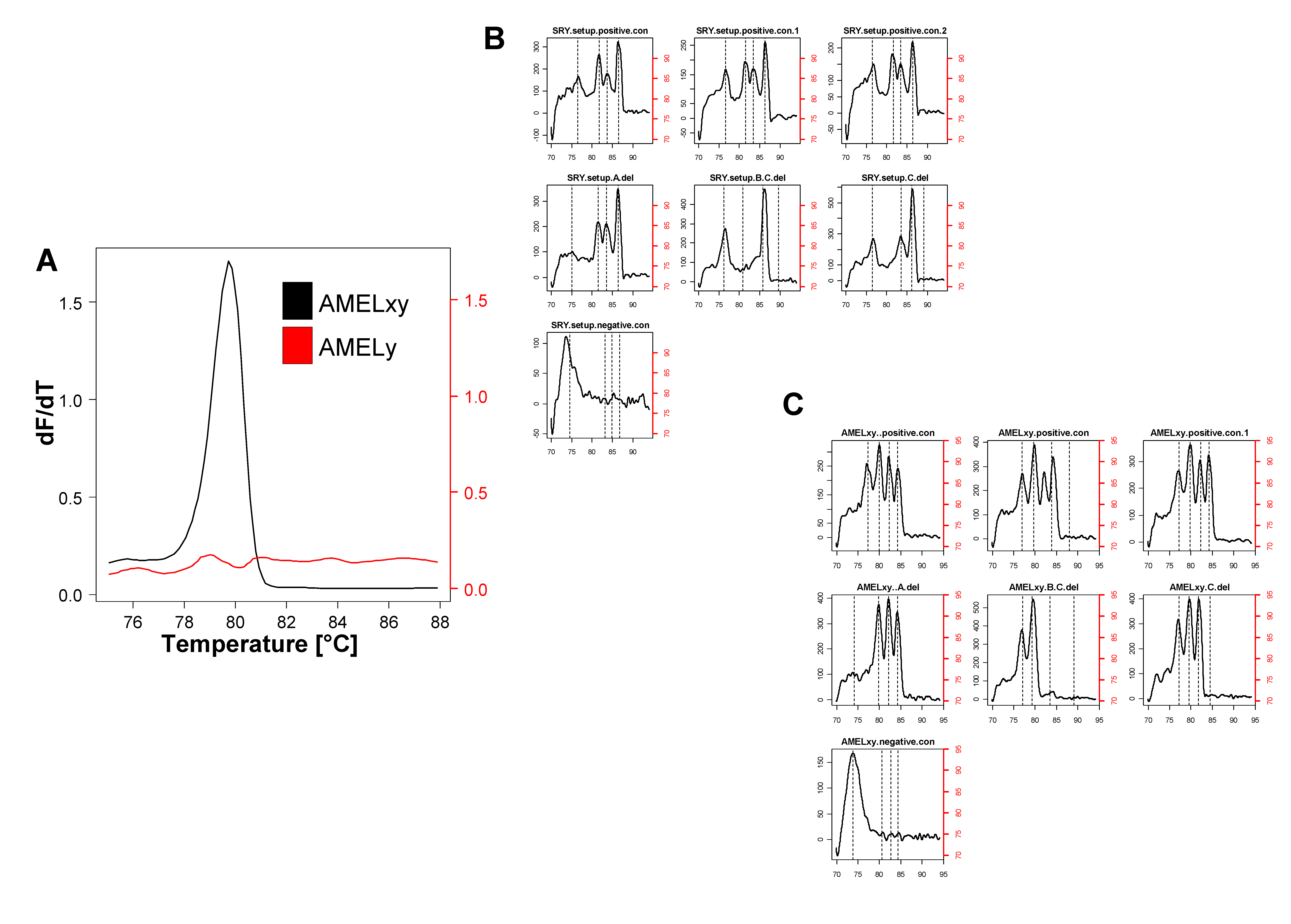

Supplement: Figure S2 — Test of new AMELxy/AMELy control primers for gender specificity and evaluation of the two multiplex setups on a block-based (96-well) quantitative PCR system (Stratagene 3000). (A) Female genomic DNA extracted from blood was subjected to quantitative PCR using the AMELxy and AMELy (alternative) primer pairs included in the multiplex setups. As expected, AMELxy gave a positive signal due to homology with the AMELx gene, while AMELy was negative. (B) and (C) Evaluation of the SRY and AMELxy multiplex setup on a 96-well quantitative PCR system (Stratagene 3000), respectively. Each of the setups was tested on three positive controls (genomic DNA from men without YCMD, upper row), samples from men with AZFa, AZFbc and AZFc-deletions (middle row) and negative control (lower row). (TIF) [file pone.0023174.s002.tif]
